# Near surface generation, burial recrystallization, and structural overprinting of carbonate platform dolomites

**DOI:** 10.1038/s41598-026-35353-4

**Published:** 2026-01-11

**Authors:** Gaurav Siddharth Gairola, Samuel T. Thiele, Pankaj Khanna, Ahmad Ramdani, Yuri Panara, Sebastian Patino, Peter K. Swart, Richard Gloaguen, Volker Vahrenkamp

**Affiliations:** 1 Energy Resources and Petroleum Engineering, Physical Science and Engineering, King Abdullah University of Science and Technology (KAUST), Jeddah, Saudi Arabia; 2 Helmholtz-Zentrum Dresden-Rossendorf, Helmholtz Institute Freiberg for Resource Technology, Freiberg, Germany; 3 Discipline of Earth Science, Indian Institute of Technology Gandhinagar, Palaj, Gandhinagar, Gujarat India; 4 MGG/RSMAS, University of Miami, 4600 Rickenbacker Causeway, Miami, FL 33149 USA

**Keywords:** Low-temperature stratabound dolomites, Cyclic dolomitization, Fracture-related diagenesis, Arab-D dolomites outcrop analog, Drone-based hyperspectral imaging, Plate-wide diagenetic signature, Stratigraphy, Sedimentology

## Abstract

Hyperspectral analysis of carbonate rocks provides a novel method to recognize not only calcite/dolomite alterations, but also to differentiate dolomite fabrics. Coupled with digital outcrop models, hyperspectral data provide an integrated representation of the geometric and mineralogical characteristics of exposed dolomite geobodies at sub-seismic resolution and over large (seismic-scale) extent. This facilitates the continuous, unbiased, and data-driven assessment of the spatial distribution of dolomites, dolomite types and properties. Here we integrate hyperspectral attributes, geochemical data, fracture analysis, tectonic, and thermal histories to constrain the process and timing of dolomitization and the compositional and textural heterogeneity at cm-scale. Our results suggest that the km-scale strata-bound dolomitized layers of the Arab-D member formed in an overall regressive system tract. near the surface (T ~ 30 °C) by refluxing of slightly evaporated seawater (-1.0 to 0‰ SMOW). With undolomitized shallow transgressive mudstone/wackestone layers forming baffles restricting downward fluid flow, the dolomitization process apparently was repetitive and linked to high frequency cycles with preferential dolomitization of cycle-top grainstone facies. Multiple reflux events during high-frequency cycle deposition led to the alternating dolomite/calcite layering. Thus, a classical one-time-dolomitize-all end-of-sequence reflux system is not indicated. The early-formed metastable dolomites were then recrystallized during burial and finally overprinted by a hot (80 °C or more) deep-seated fluid with a composition of up to 6.5‰ SMOW. This fluid was channeled by a NW-SE oriented regional fracture trend, which originated from a Late Cretaceous plate-wide structural event related to the Alpine I tectonic deformation. As the dolomite fabric was altered, porosity and permeability became enhanced. The temperatures derived from clumped isotope analysis, thermal history, and the Alpine I related fracture conduits consistently suggest a latest Cretaceous origin for the final burial dolomite maturation and textural overprinting.

**Supplementary Information:**

The online version contains supplementary material available at 10.1038/s41598-026-35353-4.

## Introduction

Sedimentary fabric, depositional facies changes, diagenetic overprints, and fracture distribution are the key factors controlling reservoir quality and associated fluid-flow heterogeneities in carbonate reservoirs^1–3^. Typically, methods for subsurface investigations lack sufficient resolution to fully resolve these factors, resulting in reservoir properties uncertainties. A fundamental uncertainty of subsurface reservoir models is the interpolation or inference of reservoir properties between wells^4,5^. This is especially challenging in carbonate reservoirs, where, due to mineralogical susceptibility, significant diagenetic modification of depositional precursors introduces variability that complicates the accurate modeling of reservoir architecture and fluid flow characteristics^6,7^. This diagenetic overprint controls both the reservoir characteristics and regulates the productivity of the depositional facies^4,8–11^. Dolomitization is a key diagenetic process in carbonate systems, capable of both enhancing and reducing permeability through mineralogical and textural modifications that directly affect reservoir storage capacity and fluid flow behaviour^12,13^. Volumetrically, dolomite contributes nearly 50% of the world’s carbonate rocks and so has a substantial impact on subsurface management plans for hydrocarbon, geothermal, and CCS/CCUS projects^14–16^.

Despite its tremendous economic importance, establishing the precise origin of dolomites and their architecture is difficult to determine, due to a lack of resolution in subsurface data and the diagenetic overprinting over time dolomites are often subjected to^17–20^. Convoluted geochemical data allow for numerous interpretations of dolomitizing fluid temperature, chemistry, and environmental conditions^18,21,22^, which rarely helps in constraining the architecture of the dolomites. In the case of platform-scale pervasive dolomitization capped by anhydrite layers, the hypersaline reflux model is generally invoked. From a geochemical standpoint, the hypersaline model is very attractive, as precipitation of anhydrite/gypsum elevates fluid Mg/Ca ratio favouring dolomite precipitation^23–27^.

One example of dolomites capped by an anhydrite cap is the Middle East’s Jubaila-Arab sequence (Arab-D reservoirs). Even though the Late Jurassic Arab-D reservoirs are one of the most prolific oil-producing formations in the world, sub-seismic interwell-scale property heterogeneities are still poorly understood. Diagenetic alteration of depositional fabrics within the reservoirs led to the development of distinct dolomite types with highly varying reservoir properties ranging from the flow baffle type to super-K layers, which are characterized by extremely high flow rates of water or oil exceeding 500 barrels per day per foot of vertical interval^8,12,28^. Low temperature origin and influence by hot fluid and temperature and textural overprinting are reported from the subsurface studies; however, the exact mechanism and timing remain uncertain^8,19,28,29^. Despite extensive studies by both industry and academia, the precise origin of the Arab-D dolomites remains unknown, as are their geometric characteristics.

Interpreting the distribution and geometry of dolomite geobodies from 1D well log and seismic data is highly uncertain due to widely spaced wells and the sub-seismic nature of dolo-bodies^13^. Outcrop analogues with laterally continuous exposed cliffs offer an opportunity to gain insights into the geometry, connectivity, and heterogeneity of dolo-bodies. However, traditional fieldwork mapping is time-consuming and interpretive, as detailed tracing of dolomites is difficult along steep cliffs. In this contribution, we employ drone-based hyperspectral imaging (HSI) to create an unbiased and continuous 3D model of a km-long outcrop that captures the vertical and lateral heterogeneities of dolomite geobodies at cm-resolution. Our results are then validated against drill cores taken directly behind the outcrop (for which hyperspectral data is also available) and synthesized with geochemical and clumped isotope data to interpret the geometry and origin of dolomites generally and within the Arab formation.

### Geological and stratigraphic framework

**Fig. 1:**
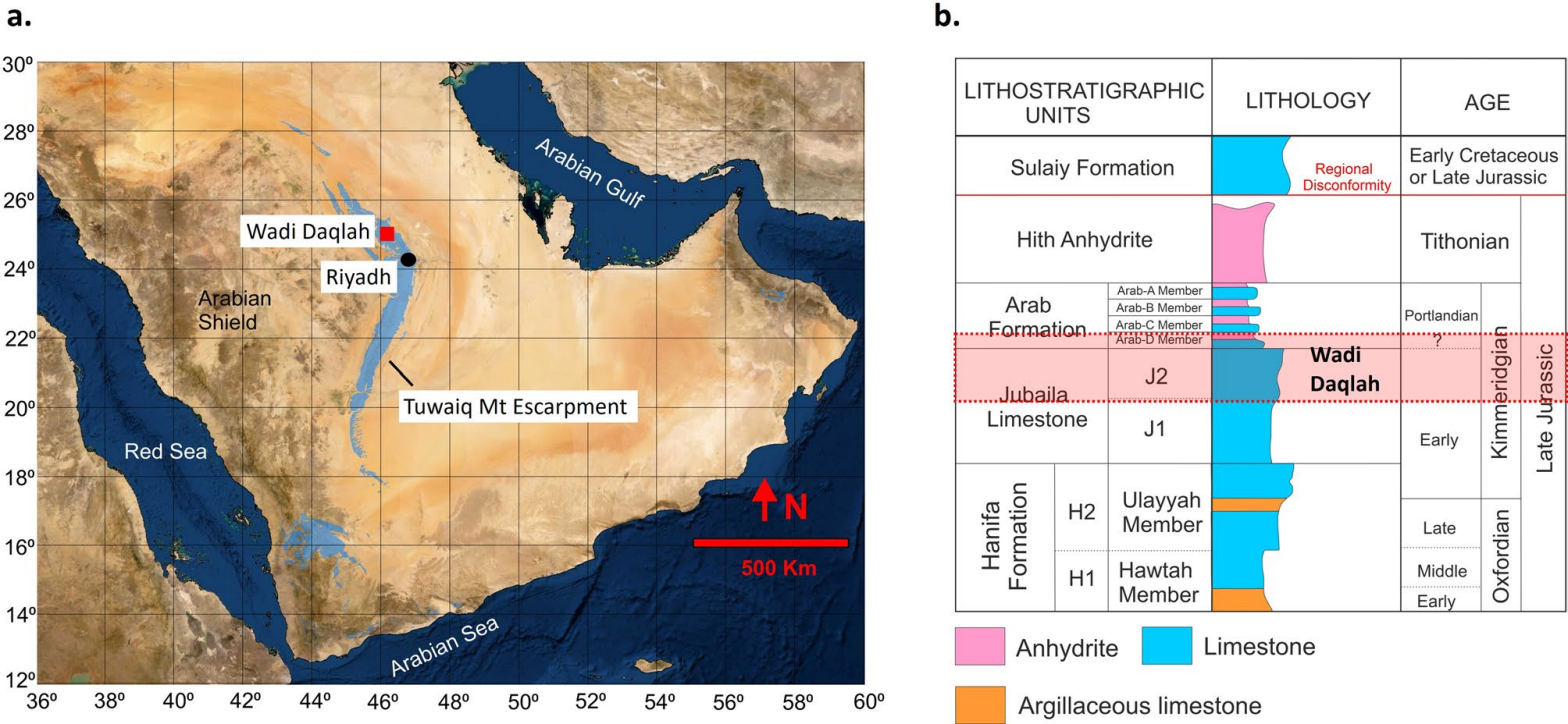
Study area and Late Jurassic stratigraphy of Saudi Arabia. **a**. Map of Saudi Arabia. (modified from^103^ showing Tuwaiq mountain escarpment (blue) (compiled from^43^with the location of study area (Wadi Daqlah) represented by red square. **b**. Generalized Late Jurassic stratigraphy of Saudi Arabia compiled and redrawn from^36,43^.

During the late Jurassic, the Arabian plate was located near and to the south of the equator under hot and dry climatic conditions^30,31^. The shelf’s northern, eastern, and southern margins were covered by an extensive (> 1000 Km) epeiric shallow sea, leading to the development of a large shallow water-carbonated platform. Several relatively deep and bowl-shaped intrashelf basins developed^30–32^, which were partially filled with high quality stacked source rock sequences^33–35^. In the meantime, in the shallow waters bordering the intrashelf basins and the Tethys margin, several sequences were deposited composed of shallow water carbonates characterized by stromatoporoid-coral boundstones, cladocoropsis grainstone/boundstone, oolitic/skeletal/peloidal grainstone/wackestone and mud dominated wackestone to packstone (interbedded with intraclastic rudstone/floatstone channels)^36–38^. These shallow water facies are the main building blocks of many of the super-giant hydrocarbon reservoirs found in the subsurface of Saudi Arabia^36,37^ and extends regionally with reservoirs also in Bahrain^39^, Qatar^40,41^ and Abu Dhabi^42^. The reservoir/source rock sequences of the Tuwaiq Mtn, Hanifa, Jubaila and Arab Formations were capped by non-porous anhydrite seals of the Upper Arab and Hith Formations (refer to Fig. 1b for the generalized late Jurassic stratigraphy of Saudi Arabia)^36,37,43^. In the subsurface, the shallow water reservoir sequences were overprinted by diagenetic alteration of the depositional fabric in addition to their depositional heterogeneities. Distinct dolomite types developed with highly varying reservoir properties ranging from flow baffles to ‘super-k’ layers, further adding complexity to the reservoir architecture and property distribution^8,13,28^.

As a consequence of the Red Sea rifting, these strata are uplifted at their western extent, with a regional dip of approximately 1° to the east^44^. The uplift and subsequent erosion of overlying strata in central Saudi Arabia led to the exposure of Late Jurassic reservoir sequences in the Tuwaiq Mountain escarpment analogous to the reservoirs further east^45–48^. Earlier studies on outcropping Arab-D reservoirs analogues focused on the Wadi Nisah area (south of Riyadh)^45–48^. However, the exposed depositional facies are not equivalent to those reported from the subsurface and contain only limited dolomite beds^45–48^.

Recently, Al Mojel et al. (2020)^43^ performed a regional study on the Late Jurassic outcrops along the Tuwaiq mountain escarpment, which indicated the presence of better reservoir-equivalent facies north of Wadi Nisah. This study focuses on an outcrop analogue at Wadi Daqlah, located 100 Km north of Riyadh, central KSA (red square in Fig. 1a), introduced first by Gairola et al., 2024^49^. The study area contains a well exposed and laterally continuous carbonate section of the Upper Jubaila Formation and overlying Arab-D member. Outcrops are capped by anhydrite dissolution collapse breccias (Arab-C). The outcrop exposes multiple stratiform dolomites with depositional and diagenetic facies similar to those reported from subsurface Arab-D reservoirs^36–38,50^ and continuous and excellent exposure along Wadi bounding cliffs for over 10 km.

## Results

### Stratigraphic framework - Wadi Daqlah

**Fig. 2:**
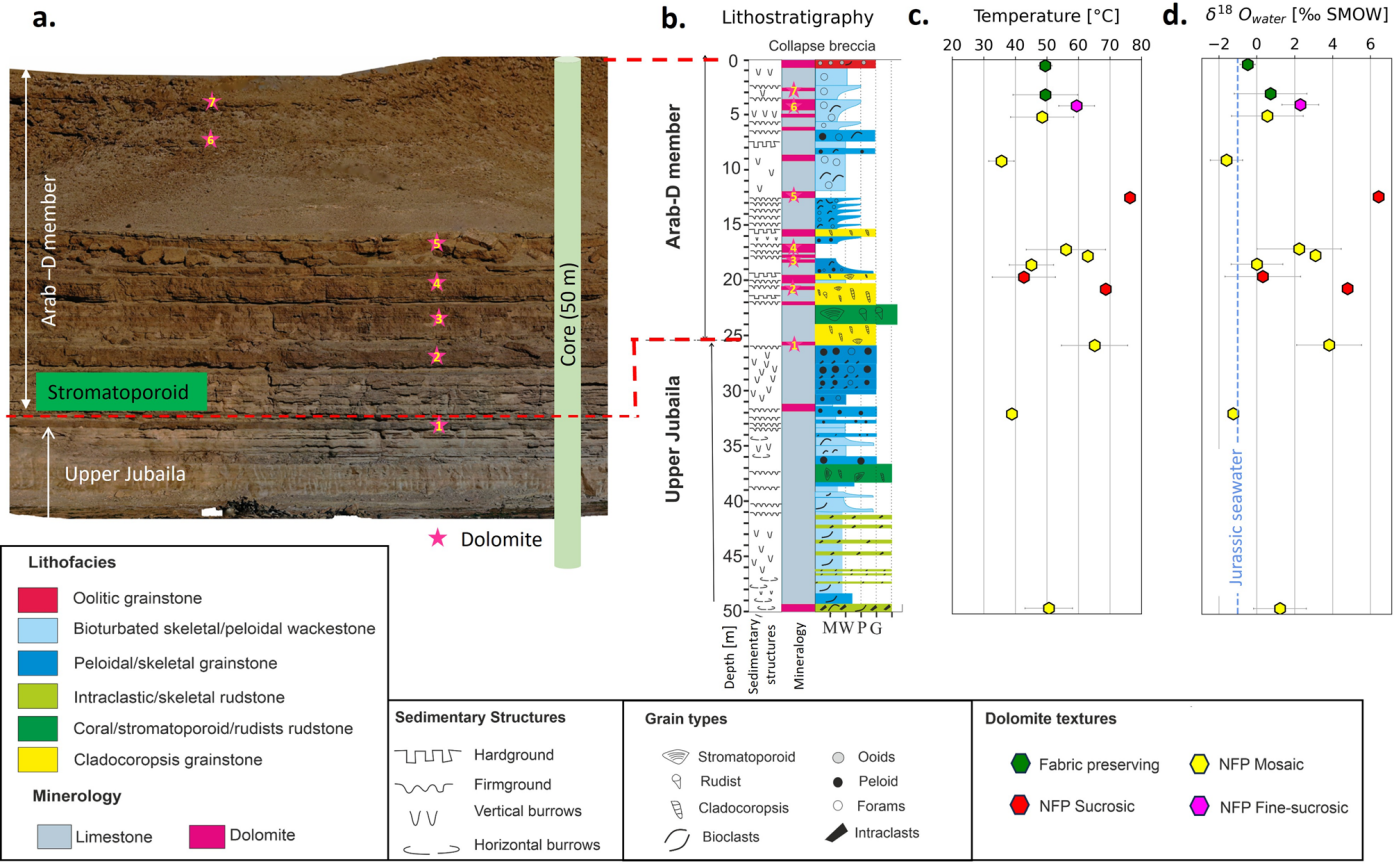
Geological framework of Wadi Daqlah: Outcrop exposure, lithostratigraphic log and clumped isotope analysis a. The figure represents the digital outcrop moded of Wadi Daqlah (**a**) showing resistant dark-brown colored multiple stratabound dolomite beds (marked by pink stars in outcrop and Lithrostratigraphic column) and generalized lithostratigraphy (**b**) of exposed facies along with clumped isotope estimated temperature (**c**) and fluid composition (**d**) with blue dashed line indicating isotopic composition of Jurassic seawater^19^.

The exposed Upper Jubaila Fm in Wadi Daqlah exhibits bioturbated skeletal/peloidal wackestone to packstone facies interbedded with rudstone channels and sheets comprising intraclasts, stromatoporoid, cladocoropsis, and corals. The Arab-D member comprises stromatoporoid/rudist rudstone to floatstone, cladocoropsis grainstone, peloidal/foraminiferal/skeletal grainstone, foraminiferal packstone/wackestone and oolitic grainstone (Fig. 2b). In addition to the depositional facies, multiple layers of resistive bed-bounded (stratiform) dolomites (indicated by the pink star in Fig. 2a) are observed in Wadi Daqlah.

### Dolomite: petrographic and geochemical signatures

Petrographic analysis indicates fabric-preserving (FP) dolomite and non-fabric-preserving (NFP) dolomite with fine-sucrosic, sucrosic, and mosaic textures which are remarkably similar to the dolomite fabrics reported from subsurface Arab-D reservoirs^8,28,36^. The δ^18^O isotope value ranges from − 4.0‰ to 0.1‰ [avg = −2.1‰], and δ^13^C isotope values range from − 0.3‰ to 2.6‰ [avg = + 1.7‰] similar to subsurface dolomites with a slight negative shift in δ^13^C values^13,28^.

Temperature estimates from clumped isotopes range from 30 °C to 80 °C, while the isotopic composition of the digenetic fluid (δ^18^O_w_) ranges from − 1.6‰ to 6.5‰ SMOW (Jurassic seawater= −1.0‰ SMOW^19^,.

### Outcrop fracture mapping

**Fig. 3:**
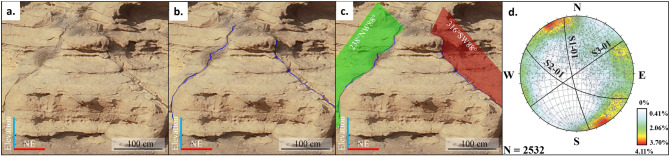
Fracture mapping workflow in 3D digital outcrop model visualized in CloudCompare software (CloudCompare 2022, https://www.cloudcompare.org/main.html). (**a**) Visualization of the 3D model, (**b**) identification of fractures and point selection, (**c**) fracture mapping and estimation of the plane of best fit, d. stereographic projections and contour plots of fracture poles showing main fracture sets plotted in Dips software (Version 6.0, Rocscience, https://www.rocscience.com/software/dips).

The fracture mapping performed on Wadi Daqlah’s digital outcrop model reveals three main fracture sets. A total of 2049 fractures were mapped. Set 1 consists of 703 fractures with an NNW-SSE orientation; Set 2 consists of 286 fractures with NW-SE orientation; and Set 3 consists of 1060 fractures-oriented NE-SW (Fig. 3). The Wadi and side-canyon systems observed in the outcrops correspond to the regional fracture sets. The main Wadi system is roughly oriented along NE-SW fracture sets, whereas side canyons are oriented along NW-SE fracture sets.

### Hyperspectral mapping

**Fig. 4:**
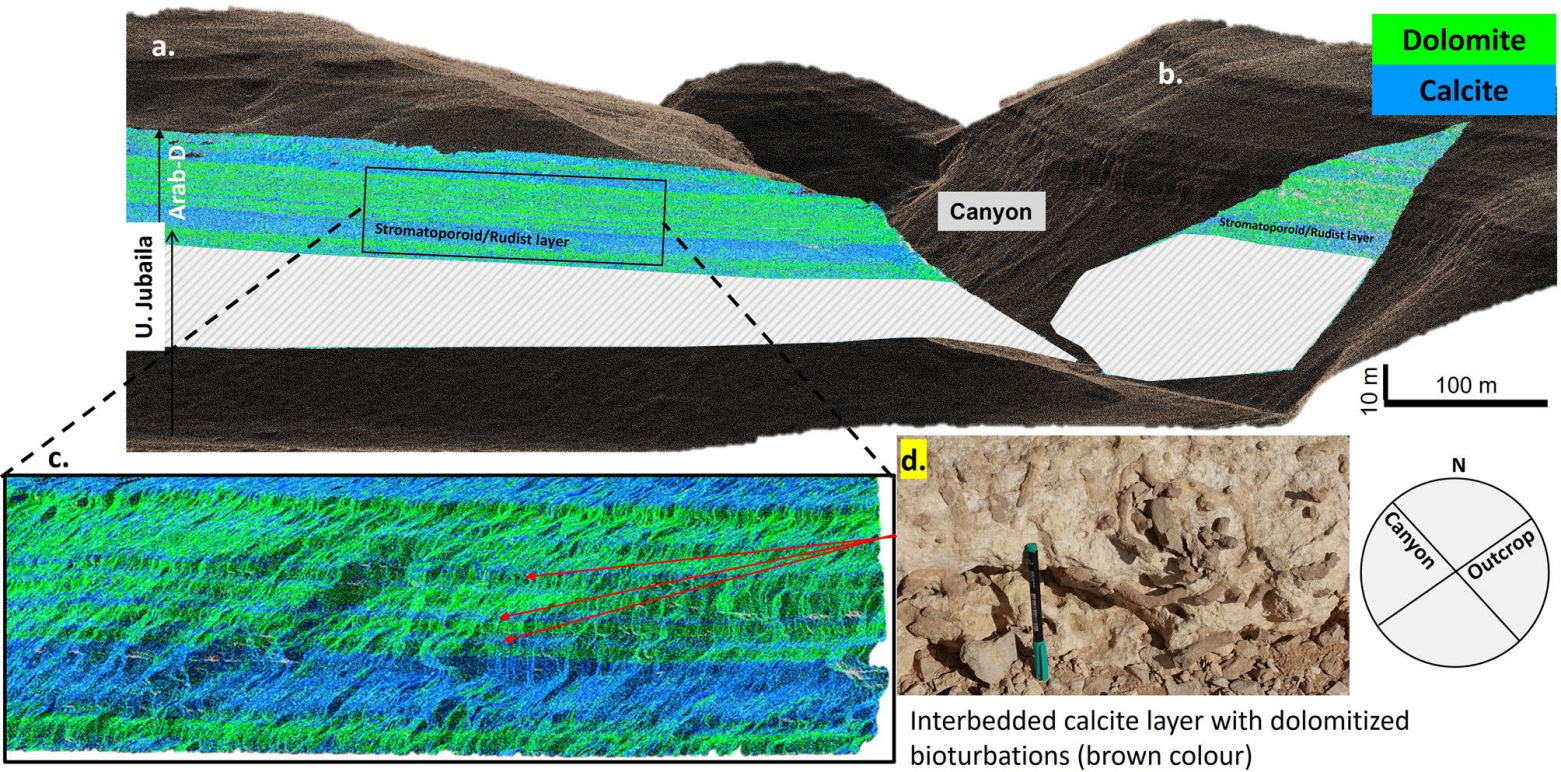
Digital hypercloud and internal architecture of dolomites. a. Digital outcrop hypercloud representing mapped carbonate mineralogy (calcite in blue color and dolomite in green) for two oucrop faces: (**a**) which is oriented NE-SW and (**b**) which is oriented NW-SE along the side canyon. The mineral mapping presents a marker stromatoporoid-rudist bed (blue color) which is overlain by stratabound dolomite layers in the Arab-D member. c. represents the zoomed-in version of the stratabound dolomites in Arab-D revealing the internal architecture with multiple dolomite layers separated by a sandwiched calcite layer. d. Field photograph of the sandwiched calcite layer with dark-brown features representing dolomitized bioturbations.

Our outcrop hyperspectral data collected using emerging UAV-hyperspectral capabilities^51^ indicates several laterally continuous dolomitized horizons that are 0.2–1 m thick and show remarkably sharp upper and lower contacts (Fig. 4a, b and c). The stratabound dolomite layers in Arab-D members are separated by thin (10–20 cm) less permeable (mudstone to wackestone) calcitic layers with dolomitized bioturbations (Fig. 4c and d).

The high spatial resolution of the HSI reveals significant dolomitization of the outcrop core with three distinct dolomitizing styles: (1) fully dolomitized horizons, (2) partially dolomitized horizons, and (3) zones with preferentially dolomitized bioturbations.

**Fig. 5:**
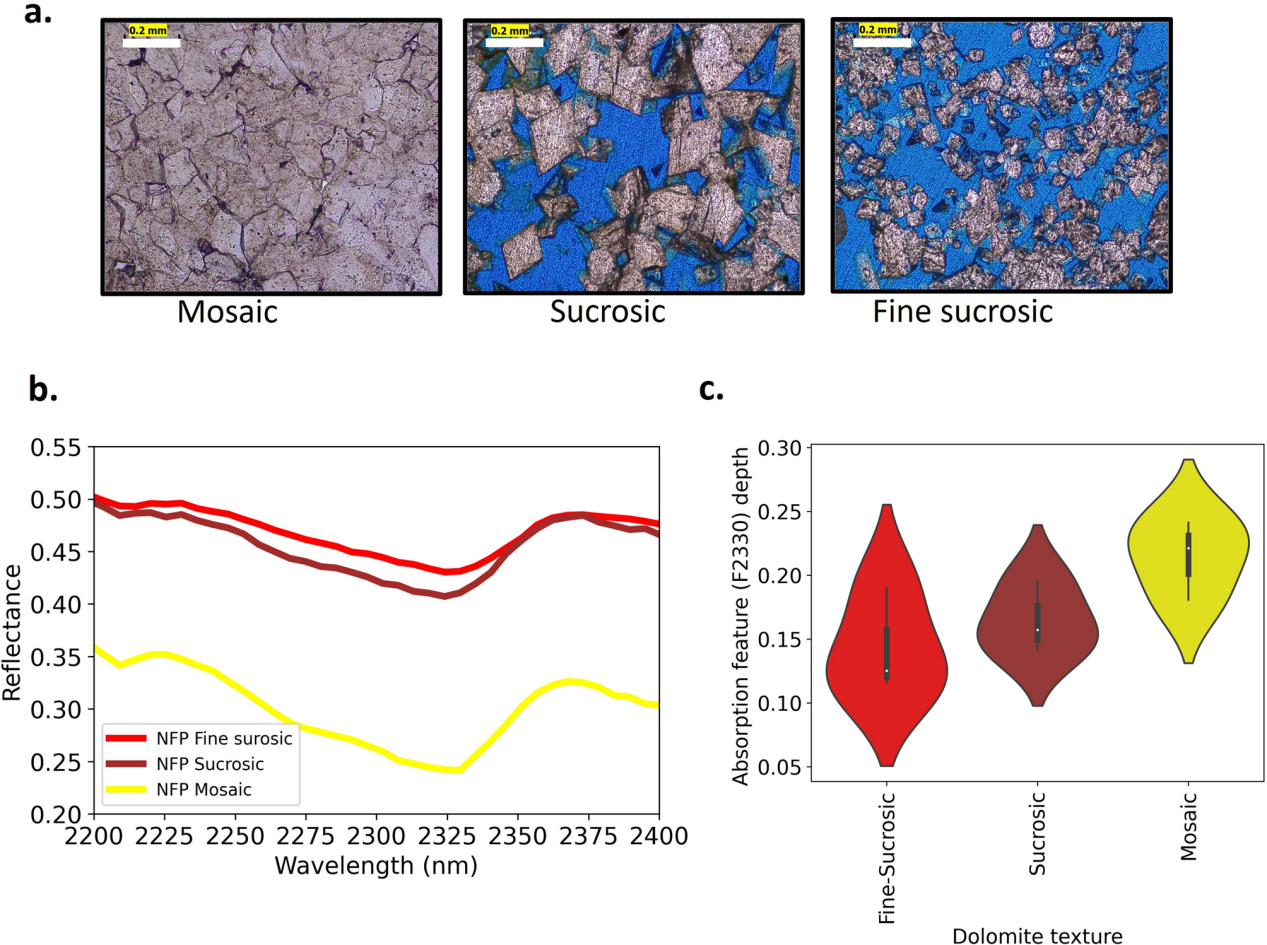
Textural end members and associated hyperspectral signatures of dolomites. a. Photomicrographs of thin sections under plane polarized light (PPL) representing textural end members of non-fabric preserving dolomites along with the representative hyperspectral signature of each dolomite texture (b). c. Represents the violin plot for depth of F2330 absorption feature for each textural member.

HSI data integrated with petrographic analysis (17 core plugs and 12 outcrop samples) reveals that fine-sucrosic (Fig. 5a) and sucrosic (Fig. 5a) textures generally exhibit higher reflectance with shallower absorption features (F2330) (Fig. 5b and c), whereas mosaic (Fig. 5a) textures have overall lower reflectance with deeper absorption features (Fig. 5b and c).This tendency is consistent with previously documented observations^52,53^.

#### Outcrop hypercloud: Dolomite-Calcite trends

**Fig. 6:**
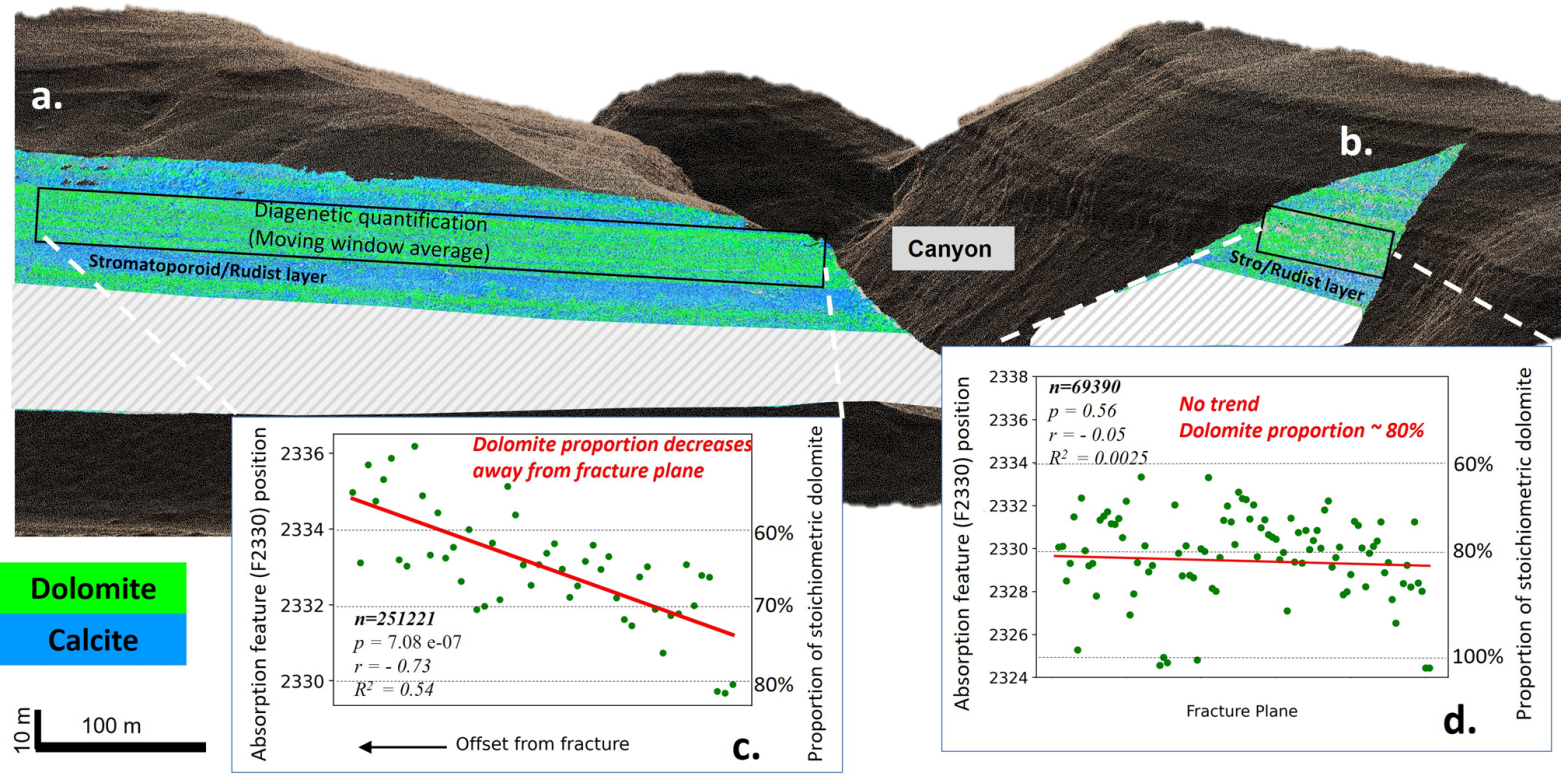
Diagenetic quantification of dolomite variability using a moving average window reveals contrasting trends with outcrop orientations. In the fracture-perpendicular outcrop (a), stoichiometric dolomite content exhibits a statistically significant decreasing trend with increasing offset from freacture, declining from approximately 80% to 60%, with *r* = − 0.73 and *p* = 7.08 × 10⁻⁷ (c). In contrast, the fracture-parallel exposure (b) shows no significant lateral variation, maintaining a consistent ~ 80% dolomite proportion throughout, with *r* = − 0.05 and *p* = 0.56 (d).

Using a moving-window average, we were able to quantify lateral variations in dolomite abundance and texture over the more than 500 m length of the outcrop. Based on the position of the absorption band of F2330 feature, the proportion of pure stoichiometric dolomite (F2330 absorption band position ~ 2325 nm) was mapped laterally on fracture perpendicular (Fig. 6a) and fracture parallel cliffs (Fig. 6b) using the correlation obtained in Gairola et al., 2024^49^. Hyperspectral data from the fracture-perpendicular outcrop (Fig. 6a) reveal a statistically significant decreasing trend in stoichiometric dolomite content, declining from approximately 80% to 60% (Fig. 6c; *r* = − 0.73, *p* = 7.08 × 10⁻⁷). In contrast, the fracture-parallel exposure shows no significant lateral variation, maintaining a consistent ~ 80% proportion throughout (Fig. 6d; *r* = − 0.05, *p* = 0.56).

#### Textural distribution of dolomites

**Fig. 7:**
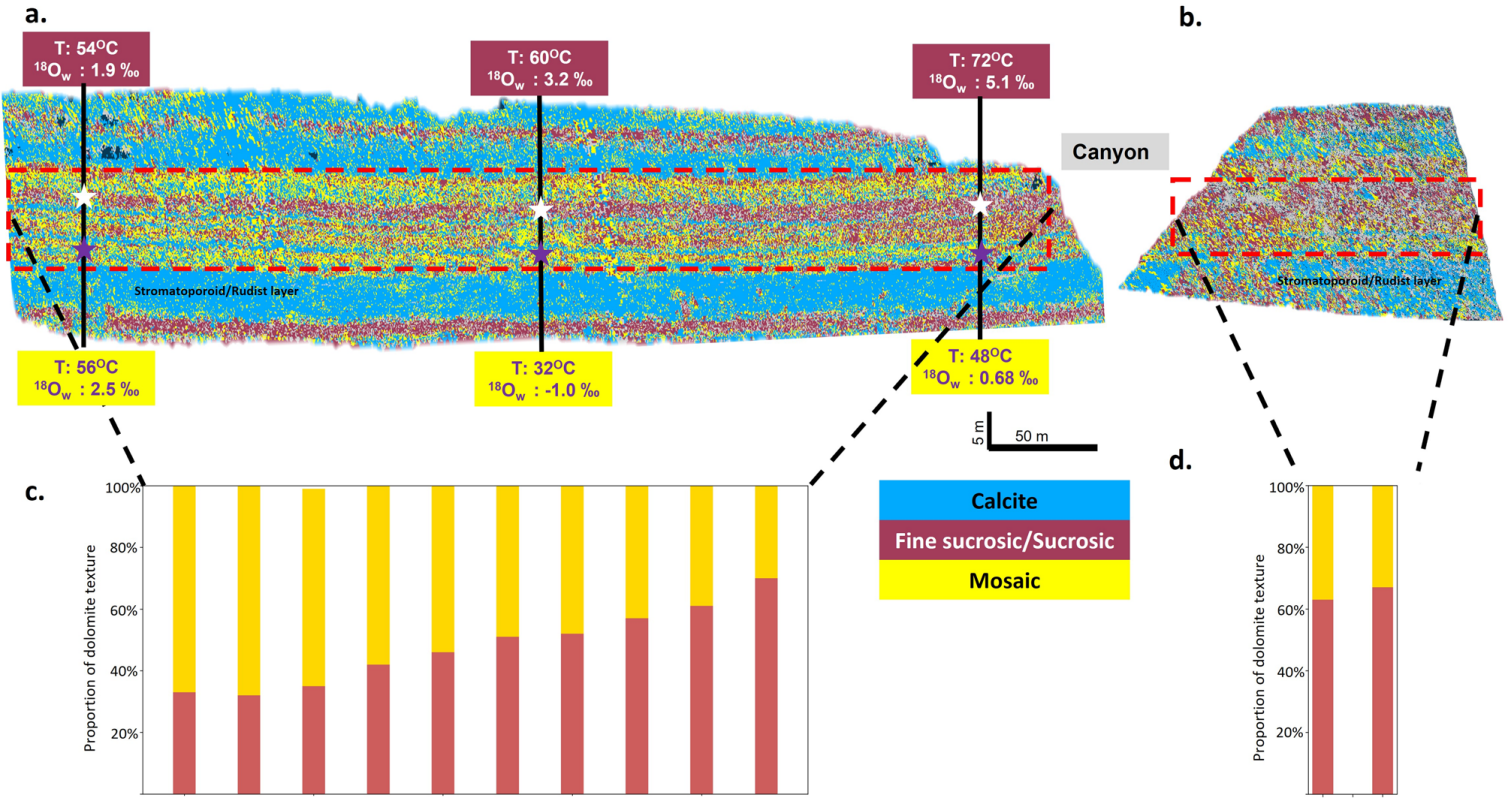
Lateral trend in dolomite texture and clumped isotope temperature estimates. Digital hypercloud showing the mapped mineralogical trend and textural variation of dolomites along with the lateral clumped isotope temperature estimates for two dolomite beds (a), showing a decreasing temperature trend is observed in the sucrosic dolomite bed (samples data in red color boxes), whereas no trend in bed comprising of mosaic texture (samples data in yellow color boxes). The histogram shows the abundance and lateral trend of dolomite texture (d). Fine-sucrosic/sucrosic texture is more prominent near the side canyon which decreases moving away from side canyon (c).

These trends are further reflected in the spatial distribution of dolomite textures. Exposure distal to the fracture zone are dominantly of mosaic texture, with select horizons exhibiting sucrosic to fine-sucrosic textures (Fig. 7a). However, moving laterally to outcrops more proximal to the side canyon, the textural distribution becomes dominantly sucrosic/fine-sucrosic (Fig. 7b). This variation is presented in the histogram of lateral distribution of dolomite texture proportions which shows the sucrosic/fine-sucrosic textures increasing from 35% to 60%; Fig. 7c). The fracture-parallel outcrop exhibits dominantly (60%) sucrosic/fine-sucrosic textures (Fig. 7d).

## Discussions

### Dolomite genesis and overprinting

Temperature and fluid composition calculations based on clumped isotopes suggest that dolomitization was initiated by slightly evaporated seawater at shallow burial depths (near the surface). Preferential dolomitization of grainstone-rich Arab-D strata and grain-rich burrows in contrasts with the wackestone/packstone-dominated sediments of the Upper Jubaila Formation and suggests that flow of dolomitizing fluids occurred preferentially along stratabound high-permeability layers. The coarse-grained sediments were preferentially dolomitized by the flow of slightly evaporated seawater during the shallowing-up phase, while the downward movement of the fluid was restricted by the low permeable underlying mudstone/wackestone layer. The presence of calcitic low-permeability baffles between stratiform dolomites indicates that the dolomitization was likely cyclic occurring before deposition of the baffling transgressive mud/wackestone layers of the next high-frequency cycle. This contrasts with a single large hypersaline reflux system associated with the formation of the overlying gypsum/anhydrite layer at the top of the Arab D or later Arab cycles which would require vertical fluid movement across baffle layers.

**Fig. 8:**
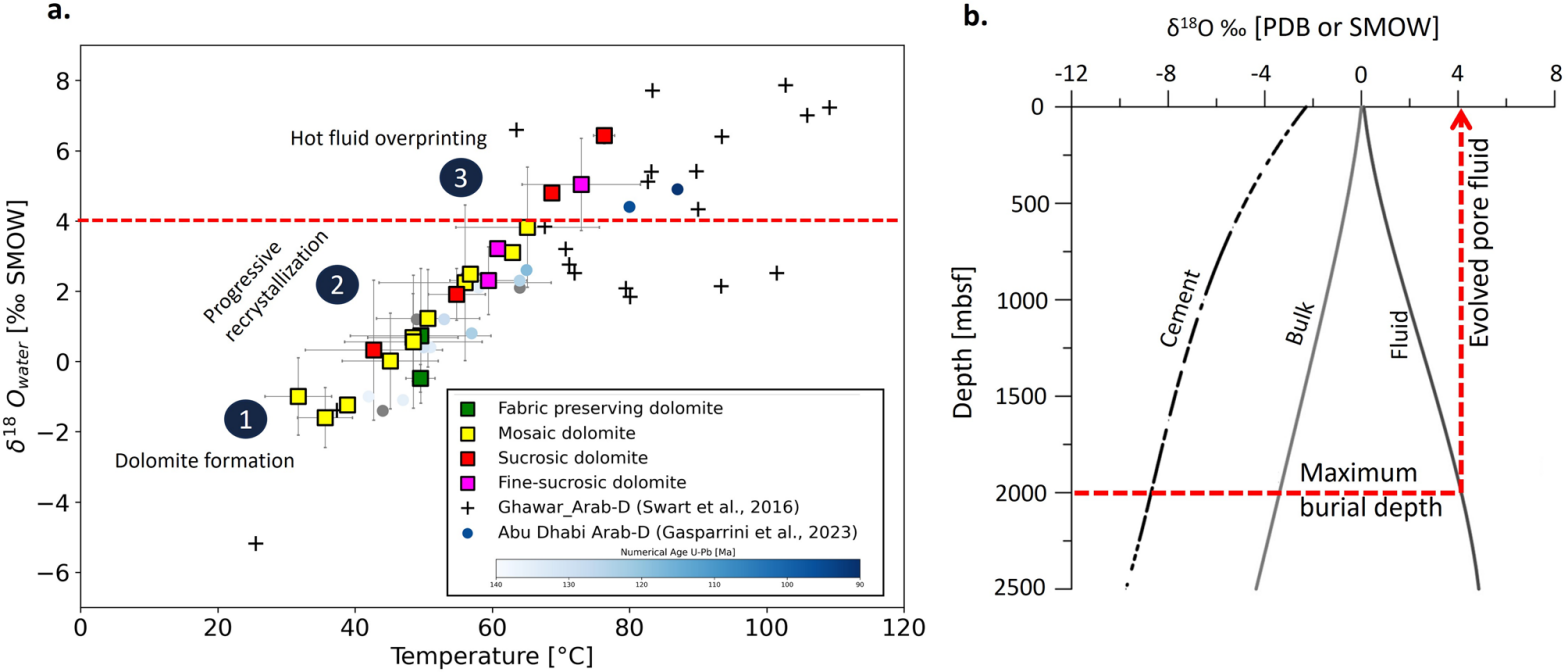
Arab-D dolomites plate-wide clumped isotope signatures and pore fluid isotopic evolution model. a. TΔ_47_ versus δ^18^O_w_ cross-plot of Arab-D dolomites. Data from this study is reported as square marker with different colors representing the dolomite textures. Data from Arab Fm. dolomites of Ghawar field, Saudi Arabia^19^ are reported with ‘+’ marker. Data from Abu Dhabi Oilfield^17^ are reported with circles, the circle color refers to the U-Pb ages (grey circles – not analyzed for U-Pb geochronology). The red dashed line represents δ^18^O_w_ cut off (+ 4‰ SMOW) for the most extreme evolved pore fluid in Arab-D based on the model presented in (b). b. Model representing expected changes in the δ^18^O value of the pore fluid and the δ^18^O value of the cement and the bulk rocks during carbonate diagenesis^19^. The red arrow indicates the most extreme evolved pore fluid isotopic composition for a burial depth ca 2000 m.

Figure 8a presents the cross-plot between (TΔ_47_) and fluid ^18^O_w_ values of Arab-D dolomites from Wadi Daqlah (data provided in Supplementary Table S1) and reported subsurface data from the Ghawar, Saudi Arabia^19^, and Abu Dhabi^17^,. The color coding in the Abu Dhabi dataset reflects the reported numerical ages obtained from U–Pb dating analysis^17^. A substantial positive correlation exists between temperature (TΔ_47_) and fluid ^18^O_w_ values, which implies recrystallization resulted from evolved pore fluid during burial. The temperature vs. fluid composition cross-plot indicates that dolomite originally precipitated at near-surface temperatures (30–35 °C) with initial fluid composition between − 1.0‰ to 0‰ SMOW, which is slightly enriched compared to Jurassic seawater (−1‰ SMOW)^19^. These early‑formed dolomites are typically metastable, characterized by disorder and non‑stoichiometric Ca‑rich compositions, and thus underwent progressive recrystallization during burial through interaction with more evolved pore fluids (as proposed in^17,20,54,56–58,58^. Recrystallization reactions enrich the fluids in ^18^O due to the preferential incorporation of ^16^O in the mineral phase at high temperatures, leaving the residual fluid with more positive δ^18^O values. Swart et al.^19^, presented a model of the evolution of the isotopic composition of pore fluid during burial with steady recrystallization (Fig. 8b). The maximum evolved pore fluid isotopic composition modeled for a depth of 2 km (maximum burial depth of Arab-D in the Ghawar field^29)^ is around + 4‰ SMOW (Fig. 8b). The maximum value observed in the studied samples is6.5‰ SMOW, which indicates influence by a deep-seated fluid with much more evolved pore fluid.

**Fig. 9:**
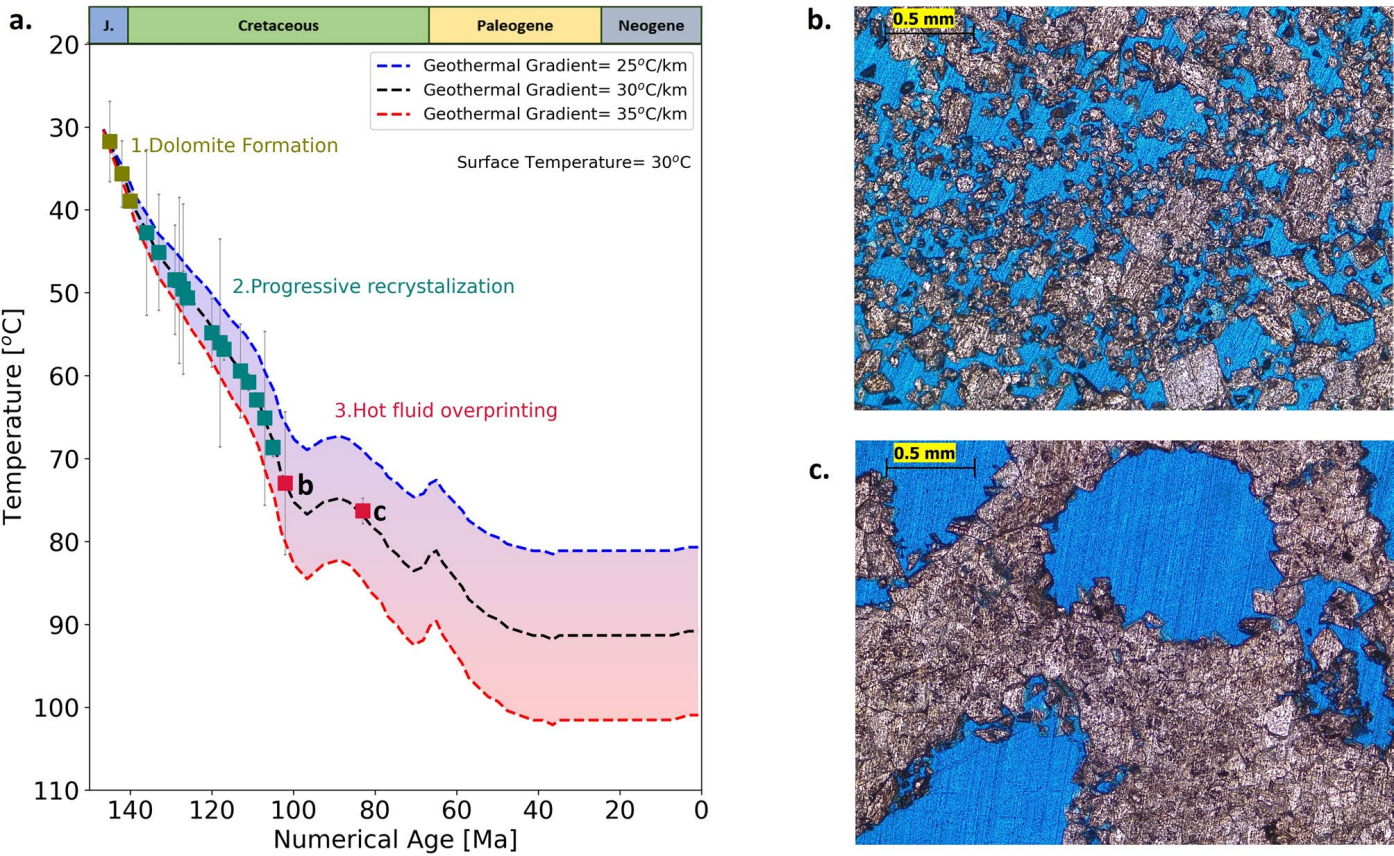
Thermal history model with clumped isotope temperature overlay. (a) Modeled thermal histories of the Upper Jurassic Arab Formation from the Ghawar Field^29^ were reconstructed by considering different geothermal gradients. The colored squares represent the temperature estimates from clumped isotope analysis indicating three stages: dolomite generation, progressive recrystallization, and hot fluid overprinting. (b) and (c) represent the thin section photomicrographs of the samples highlighted by ‘b’ and ‘c’ in ‘a’ respectively.

Clumped isotope values indicate a wide range in temperatures and associated fluid compositions: Fabric-preserving dolomite [Temperature: 49.5 to 49.6 °C (m: 49.55 °C); δ ^18^O_w_ : −0.4 to 0.7‰ SMOW (m: 0.13‰ SMOW)]; Mosaic dolomite [Temperature: 32 to 65 °C (*m: 49.55*^*0*^*C*); δ ^18^O_w_ : −1.6 to 3.8‰ SMOW (m: 0.93‰ SMOW)]; Sucrosic dolomite [Temperature : 42 to 76 °C (m: 60.64 °C); δ ^18^O_w_ : 0.3 to 6.5‰ SMOW (m: 3.36‰ SMOW)]; Fine-Sucrosic dolomite [Temperature: 60 to 72 °C (m: 64.4 °C); δ ^18^O_w_ : 2.3 to 5.04‰ SMOW (m: 3.52‰ SMOW)]. There is a wide range and significant overlap in clumped isotope-derived estimates across different dolomite types. This complicates the reconstruction of a clear pathway for textural origin and evolution. However, when considering the mean values for estimated temperature and isotopic composition (salinity) of the dolomitizing fluid, increasing temperatures and salinities are observed from fabric-preserving/mosaic to mosaic, and then to sucrosic textures. The most extreme temperatures and δ^18^O_w_ are associated with dolomites exhibiting dissolution features with higher porosities (Fig. 9a and c) which may be due to leaching of metastable dolomite or calcite by hot fluid.

### Fracture system - overprinting pathways

Since the deposition of the Jurassic sequence, the region has been involved in two main tectonic phases related to the closure of the Neo-Tethys Ocean^59^. The first, named Alpine I, occurred during the Late Cretaceous (Turonian to Maastrichtian) following at least two phases of an oblique collision between the Indian and the Arabian Plate^59^. For the Arabian plate, the key Alpine I structural deformation event leading to fracture generation is associated with a collision/Ophiolite emplacement during the Santonian to Maastrichtian (the Masirah event) which resulted in a maximum horizontal stress-oriented NW-SE^60,61^. A second phase of tectonic deformation, named Alpine II, occurs later in the late Oligocene-Miocene. This phase is characterized by a NE-SW oriented maximum horizontal stress, resulting from the collision between the Arabian and Eurasian plates^60,62^. Detailed fracture analysis performed on the same outcrops indicates three prominent fracture sets with NW-SE, NE-SW, and NNW-SSE orientation. These fracture sets are observed all along the Arabian plate and are related to the major tectonic events Alpine I and Alpine II^60,63^. The outcrop hypercloud depicted in Fig. 4a is oriented roughly northeast–southwest (NE–SW), whereas the side canyons align with a northwest–southeast (NW–SE) regional fracture trend^60,63^. The textural mapping indicates the dominance of fine-sucrose texture (leached dolomites) near the side canyon. Also, a temperature gradient exists, as evidenced by the clumped isotope results obtained from the lateral outcrop samples of the fine-sucrosic dolomite bed (Fig. 7a, temperature in red box). No trend is observed in the mosaic dolomite bed (Fig. 7a, temperature in yellow box). This alignment suggests that hot fluids flowed through a fracture system at depth, entered the already dolomitized layers, overprinted the early dolomites and their geochemical signature.

### Burial history: timing of recrystallization

**Fig. 10:**
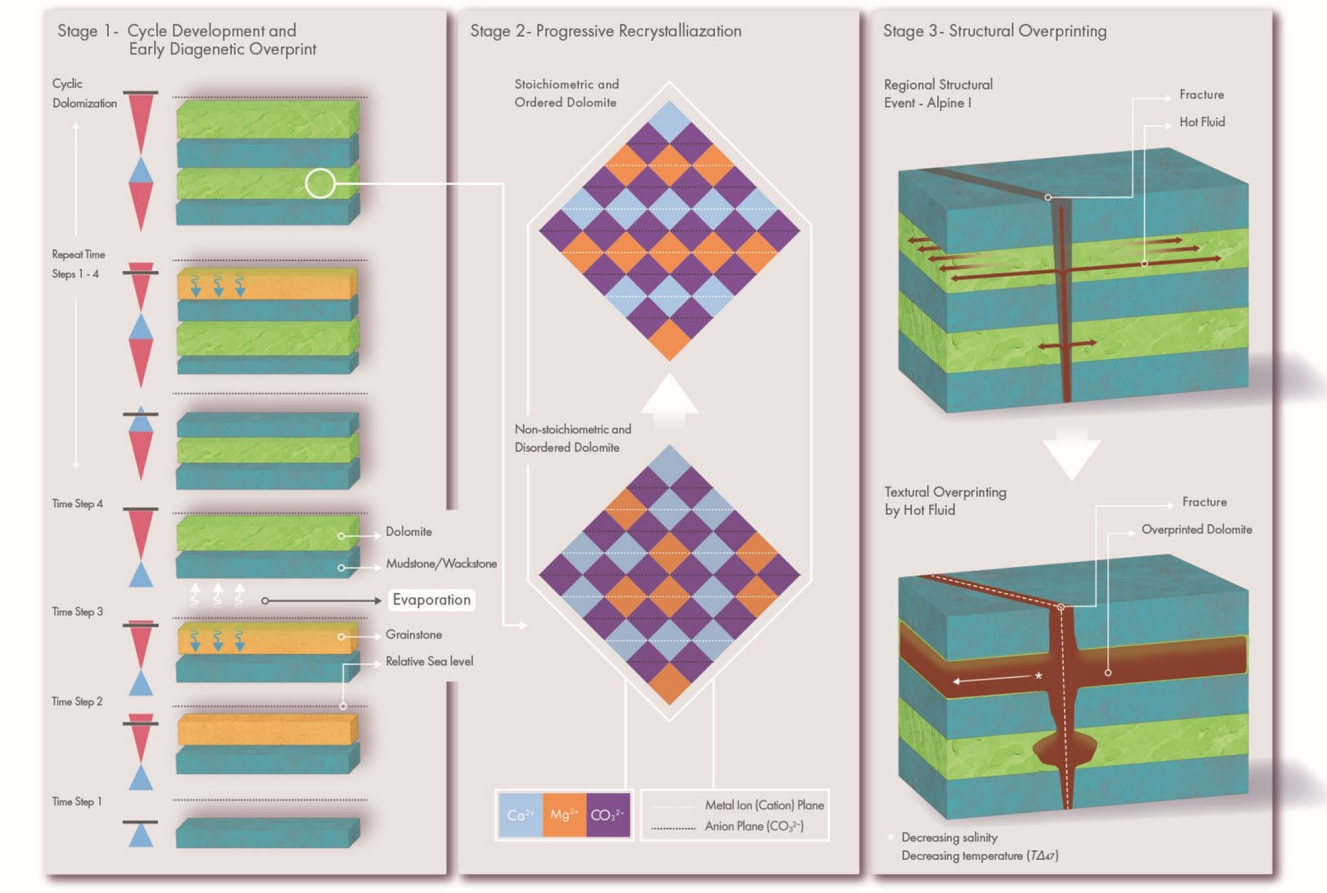
Dolomite diagenesis: A conceptual three stage model. The figure illustrates the diagenetic evolution of stratabound dolomites, showing cyclic dolomitization of cycle top grainstones facies in response to shallowing-up conditions during high frequency cycles (Stage-1), followed by progressive recrystallization of early formed metastable (non stoichiometric and disordered) dolomites to more stable (more stoichiometric and ordered) dolomite under burial (Stage-2), and late-stage hot fluid overprinting assisted by NW-SE oriented Alpine I related fracture sets (Stage-3).

The Jubaila-Arab sequence was deposited in the Late Jurassic, followed by progressive burial until the onset of the first compression event in the Late Cretaceous, which induced fracturing, faulting, folding, and minor uplift. This event was followed by further subsidence, with the maximum burial depth in the Ghawar field around 2 km^29^. The presence of well-developed stylolites observed in the cores and outcrops of Wadi Daqlah attests to the fact that this location has also experienced substantial burial. Based on the estimated burial depth evolution of the Arab formation^29^, we have modeled thermal histories (Fig. 9a, data provided in Supplementary Table S2) based on different geothermal gradients between 25 °C/km and 35 °C/km (present-day geothermal gradientis 28 °C/km^64^. The resulting thermal history curves are shown in Fig. 9a, along with clumped isotope-derived temperature estimates for the samples. Interpretation of the clumped isotope data from the thermal history plot (Fig. 9a) indicates that dolomitization initiated at near-surface temperatures (represented by olive-colored data points in Fig. 9a), followed by progressive stages of dolomite formation (teal-colored data points in Fig. 9a). This progressive recrystallization a suggests that early formed dolomites were likely metastable, characterized by non-stoichiometric and disordered crystal structures which subsequently undergo burial recrystallization, resulting in more stable, stoichiometric, and ordered dolomite. This diagenetic transformation pathway is illustrated as Stage 2 in Fig. 10. The latest dolomite maturation event (red-colored data points in Fig. 9a) occurred in the Late Cretaceous (Fig. 9a) at the same time as the initiation of Alpine I event and the creation of NW-SE fracture sets.

### Conceptual model of dolomitization and dolomite diagenesis

Based on the integrated results from clumped isotopes, textural distribution, burial history and petrographic analysis, the following three stages of dolomite paragenesis (Fig. 10) are proposed:

#### Stage 1

Temperature and fluid composition estimates from clumped isotopes suggest that stratabound dolomite layers in the Arab-D member likely formed near the surface (30 °C) by slightly evaporated seawater (−1 to 0‰ SMOW). During an overall shallowing upwards trend of a larger scale cycle, the dolomitization process was cyclic, with dolomitization of grainstone-rich facies during the regressive stages of high-frequency cycles. Mudstone/wackestone layers which constitute the shallow transgressive part of the high-frequency cycles are of low permeability. They formed baffles and hence resisted dolomitization by the refluxing brines. Only grain-filled burrows in the mudstone are also commonly dolomitized. Repetitive high frequency cycles of shallow transgressive mudstone/wackestone and regressive grainstone units led to a stack of interbedded layers of limestones and strata-bound dolostones (similar dolomitization style is reported from Cretaceous carbonates in Central Texas^65^. This repetitive reflux dolomitization is a permutation of the classical end-of-cycle reflux model that acknowledges the baffling nature of the mudstone layers and the generation of alternating limestone/dolostone layers without invoking vertical communication across all layers.

#### Stage 2

Early‑formed dolomites are generally metastable, characterized by a disordered ionic arrangement and Ca‑rich non‑stoichiometric compositions^54,56,58,58^. These early-formed metastable dolomites were subsequently recrystallized by evolved pore fluids (enriched ^18^O values) during burial. The recrystallization affected the dolomite heterogeneously, with only some of the dolomites preserving the original low temperature and fluid composition signature.

#### Stage 3

Finally, the dolomites were overprinted by thermally convecting deep basinal fluids ascending along fractures at a temperature of 80 °C or more. These highly overprinted dolomites dominantly have leached and moldic features (Fig. 9b and c) exhibiting enhanced porosity. Similar results were reported from subsurface Arab-D reservoir in the Ghawar field^19^, suggesting leaching and recrystallization with ascending hot fluid. While these fluids may have originated from different depths and involved distinct reaction pathways, their association with leached dolomite textures implies undersaturation with respect to calcite and meta-stable dolomite. Based on our observations of compositional gradients away from fractures associated to a regional tectonic event, vertical fluid migration was facilitated by a fracture system. However, the effective sealing of the overlying anhydrite layer (Arab-D anhydrite; Fig. 1b) necessitated the lateral migration of the fluids into the permeable dolomite horizons (similar to the mechanism proposed in^66^. The mineralogical and textural patterns, the clumped isotope-derived temperature trends perpendicular to the NW–SE fracture trend, indicate a Late Cretaceous age for diagenetic overprinting associated with Alpine I regional tectonism. Late phase overprinting of dolomites apparently occurred concomitantly further eastwards in Abu Dhabi based on latest Cretaceous U-Pb based age estimates of Arab-D dolomite from a reservoir^17^.

## Conclusions

Our continuous and high spatial resolution characterization of outcrop, mineralogy and texture allow us to constrain the geometry and spatial relationships of dolomite geobodies. These results imply that:

Combining field-calibrated hyperspectral scans of km-scale outcrops, detailed petrography, estimates of temperature and diagenetic fluid compostion derived from clumped isotope analysis in combination with a burial/geothermal history curve offers critical insights into dolomite formation processes and the timing of diagenetic dolomite maturation.

Clumped isotope thermometry constrains the temperature of dolomitization from 30 °C to 80 °C and the composition of dolomitizing fluids from − 1.6 to 6.5‰ SMOW. Temperature and salinities shows relationship with dolomite textures with the most extreme temperature and salinities are associated with sucrosic/fine-sucrosic dolomites textures exhibiting leaching and moldic features with enhanced porosities.

High frequency cycles composed of shallow trangressive mudstone/wackestone and regressive grainstone layers in the overall regressive Arab-D setting created an environment conducive to repetitive early-diagenetic reflux dolomitization in laterally continuous and extensive grainstone layers. Early dolomites were likely metastable. Low permeable mudstone/wackstone layers remained calcitic.

Progressive burial and increasing temperatures led to the stabilization of early formed metastable dolomites.

The generation of a regional fracture system associated with the Alpine I tectonic event led to fracture assisted convective flow of deep-seated fluids entering dolomitized layers. This caused the leaching of metastable dolomites or calcites increasing porosity and permeability especially near fractures.

In reservoirs, km-scale lateral extension of multiple stacked stratabound dolomites with contrasting textural characteristics, separated by thin mudstone/wackestone baffle layers creates zones of contrasting reservoir properties. This architectural complexity will cause inter-well flow heterogeneity, which would significantly affect reservoir performance. Recognizing the impact of fracture patterns on dolomite diagenesis provides insight into the timing of burial fluid circulation and the potential to constrain reservoir property heterogeneities.

The integration of hyperspectral attributes from outcrop imaging with geochemical data, fracture characterization, and petrographic analysis offers robust spatial constraints and enables reconstruction of dolomitization timing and mechanisms, thereby supporting the development of more realistic subsurface models.

## Materials and methods

This study focuses on an outcrop analogue in Wadi Daqlah, located 100 Km north of Riyadh, in central Saudi Arabia, first introduced in Gairola et al., 2024^49^. The datasets include drone-based photogrammetry (conventional RGB and hyperspectral), 50 m long behind the outcrop core, and outcrop samples (*n* > 100). The core plugs were drilled every 30 cm, and the trimmed ends of the plugs were utilized to prepare thin sections and powder samples for geochemical analysis.

### Digital outcrop capture and analysis

Photogrammetric models of these outcrops were captured using a DJI Phantom 4 RTK quadcopter with an average distance of 50 m from the outcrop. On average, 2000 high-resolution images were acquired using a 20-megapixel camera (up to 2 cm per pixel resolution) with an 80 to 95% overlapping. In addition, an onboard Real Time Kinematic (RTK) GPS automatically georeferenced the images with a theoretical accuracy of 0.1 m (horizontal) and 0.15 m (vertical). The drone dataset was processed using Agisoft metashape (version 1.7, https://www.agisoft.com) as per the methodology described in Khanna et al., 2020^67^. The Digital outcrop model (DOM) was then utilized for 3D fracture mapping as per the methodology described in^63,68,69^. This 3D approach efficiently visualizes fractures (and bedding) along their visible extensions, resulting in robust identification of fracture sets and quantifying fracture intensity across the outcrop. We manually mapped 3D fracture traces with open-source software CloudeCompare (CloudeCompare, 2022, https://www.cloudcompare.org/main.html) by picking points along individual fracture traces (Fig. 3b) and subsequently constructing 3D planes that best fit a given point selection (Fig. 3c). We mapped fracture traces with uninterrupted exposure using the computer-aided manual mapping tool, the ‘Trace tool’ of the Compass plugin^70^, which allowed us to semi-automatically draw fracture traces between two points defined on fracture traces or bedding.

### Hyperspectral data acquisition and correction

Subsequently, a HySpex Mjolnir VNIR-SWIR hyperspectral camera was flown on a custom-built UAV to map key outcrops within Wadi Daqlah. Hyperspectral data was acquired obliquely to minimize spatial distortion on the cliff faces, and the UAV was flown at a distance of ~ 50 m from the cliff face to achieve a ground-sampling distance of 2 cm per pixel. The raw hyperspectral data was then converted to radiance using sensor-specific software provided by HySpex and geometrically corrected by back projection on the dense photogrammetric point cloud using the method described by Thiele et al., 2022^51,71^. This radiance hypercloud was then converted to estimated reflectance using data from sunlight and shaded calibration panels acquired before and after each flight (while the UAV was on the ground), also following the methodology described by Thiele et al., 2022^51^.

Hyperspectral drillcore data was acquired using a Specim SiSuROCK core scanner and converted to reflectance using the empirical line correction method, described in depth by Gairola et al., 2024^49^.

### Hyperspectral data analysis

The resulting reflectance hyperclouds and hyperspectral drillcore data were then analyzed using the hylite software package^71^. The minimum wavelength mapping technique^72,74,74^ was used to map the carbonate feature (F2330) depth and position (2325–2345 nm) across the outcrop. This feature is caused by vibrational overtone processes of carbonate ion bonds (CO_3_^2−^), and its position can be used to distinguish dolomite (~ 2325 nm) from calcite (~ 2345 nm)^51,75,77–84,84^. The outcrop 3D-hypercloud was visualized in CloudCompare software (CloudCompare 2022, https://www.cloudcompare.org/main.html).

Dolomite textural variations were derived from the depth of the carbonate absorption feature which is sensitive to grain size and texture^52,53^. Figure 5b reveals that fine-sucrosic and sucrosic textures generally exhibit higher reflectance with shallower absorption features (F2330), whereas mosaic textures have overall lower reflectance with deeper absorption features to systematically classify these textures across the hyperspectral dataset, we applied a decision tree analysis to the hypercloud, enabling automated classification based on both mineralogy and dolomite texture. The first pass is based on the position of F2330 feature, which distinguishes dolomite and calcite using a threshold of 2335 nm. Once dolomite spectra were isolated, further textural end members within the dolomites were mapped using the MWL-derived depth of the F2330 feature with a threshold value of 0.18. The threshold was established based on the violin plot of depth of feature F2330 as shown in Fig. 5c which was derived by representative thin-section analysis from the core (Fig. 2) and precisely located outcrop samples (Fig. 7a). Among the dolomite textures, the defined classes are mosaic and sucrosic/fine-sucrosic based on the spectral signature. The FP dolomite is not considered for mapping as the outcrop does not expose the FP dolomite, which occurs only at the top, just below the collapse breccia.

### Petrography and geochemical analysis

In addition, a detailed petrographic and geochemical analysis was performed on the core and outcrop samples to obtain detailed maps of mineralogical variation, calibrate hyperspectral signatures, and guide future sampling strategies. More than 200 thin sections were prepared from core and outcrop samples which were dual-stained (Alizarin red and Potassium Ferrocyanide) to facilitate the differentiation of dolomites from calcite and ferroan and non-ferroan dolomites, respectively^85^. These stained sections were analyzed using hyperspectral imaging, X-ray diffraction (XRD), and petrographic techniques to identify candidates for subsequent isotope analysis. Aiming to reveal the timing and process of dolomitization, a subset of 60 samples were analyzed for O-C isotope, out of which 20 samples (14 from core and 6 lateral samples from outcrop) were selected for clumped isotope analysis performed at the University of Miami as per the methodology described in the following subsections:

#### Carbon and oxygen isotopes

Approximately, 5 mg of powder from each sample were weighed into individual copper sample boats and loaded into a Fairbanks auto sampler device. Samples were reacted in phosphoric acid using the common acid bath method. Produced CO_2_ was analysed on a Finnigan MAT 251 mass spectrometer. Samples were corrected to a Carrara standard and data are reported relative to Vienna Pee Dee Belemnite (VPDB) scale as defined for carbonates by the δ^13^C and δ^18^O values of NBS-19^86^.

#### Clumped isotopes

The methodology used for clumped isotope analysis is similar to that described by Smith et al. 2022^87^. Each sample was homogenized, and approximately 7 mg was used for each analysis. Samples were prepared on a vacuum gas extraction line and reacted in phosphoric acid (1.93–1.95 g/cc) in a 90 °C common acid bath for 30 minutes. To remove water, non-CO_2_ gasses, and organic matter from the sample, the produced CO_2_ gas was passed through a series of U-traps and a Porapak™ Q trap cooled with methanol slush. The cleaned CO_2_ gas was fed into a dual-inlet Thermo-MAT253 isotope ratio mass spectrometer, with the mass-44 beam intensity set to 12 V. The sample gas and the in-house reference ‘working gas’ volumes were balanced between bellows, and measurements of sample and standard gas were taken alternately, with the standard gas calibrated to the NBS-19 standard. Using the pressure baseline method, six blocks of analysis were run per sample, with 14.5 alternations between the reference and sample gases, and beam intensities ranging from 44 to 49 were measured. The integration time was 14 seconds, with a 15-second delay before starting the measurement. Each block has four ‘off peak’ measurements at the start and end, as well as sixteen ‘on peak’ measurements in the middle. The negative pressure baseline (PBL) on mass 47 was measured during the ‘off peak’ analysis, followed by a PBL correction^88^. The total integration time for peaks was 2521 s. Merritt and Hayes’ (1994)^89^ calculations yielded a shot noise estimate of 0.008‰ for Δ_47_ at 12V. Data normalization and calculation of raw Δ_47_ values followed Huntington et al.‘s^90^ framework, with additional recommendations from Daëron et al.^91^.

Dennis et al.‘s^92^ approach and Bernasconi et al.‘s^93^ Intercarb CDES correction were used to convert raw Δ_47_ values to the carbon dioxide equilibrated scale (CDES). Standardized CO_2_ gases were equilibrated with internal laboratory standard waters at 25 °C and 50 °C for 48 h, followed by several hours of equilibration at 1000 °C to produce carbon dioxide standard gases for CDES correction. Murray et al.^94^ provide a thorough analysis of the standard preparation methods. Every 20–30 days, standard equilibrated gases were processed and analysed. To account for sample drift during the run, the slope and intercept of δ^47^ and ∆_47_ values were calculated and linearly interpolated.

The full carbonate ETH standard set (ETH-1, 2, 3, and 4) was processed weekly to correct ∆_47_ values for I-CDES. Values were averaged over a three-week period, which roughly corresponds to the time when the equilibrated gases were measured. A transfer function based on the measurement of the four ETH standards was employed^93,95^. During each measurement period, ~ 40% of all carbonates analyzed were either carbonate standards or equilibrated gases. As both correction methods can be considered statistically identical, this study uses the CDES framework. The δ^13^C and δ^18^O values on the samples that were subjected to clumped isotopic analysis are reported relative to VPDB as described previously. No acid fractionation factors were applied to the clumped isotopic data^96^, and temperatures were calculated using a formula developed by Swart et al.^97^ for reaction at 90 °C.

#### Calculation of δ^18^O fluid values

Multiple calibration equations have been proposed for temperature estimation in dolomites, differing in slope and resulting values^19,98,100,101,101^. In this study, we adopt the methodology of Swart et al.^19^ to enable a direct comparison between outcrop Arab‑D dolomites examined here and subsurface Arab‑D dolomites reported in that work. The δ^18^O_fluid_ values of the fluids responsible for the precipitation of the dolomites were calculated from the measured clumped isotope-derived temperatures (as described in^19^ and the δ^18^O values measured during the clumped isotope analysis using equation by Horita^102^.

## Supplementary Information

Below is the link to the electronic supplementary material.

Supplementary Material 1

## Data Availability

The hypercloud datasets will be made available upon request from the corresponding author (GSG), and all other data is available within the manuscript.
